# Caries experience and social insertion in schoolchildren from an urban population in the ecuadorian highlands: a descriptive study

**DOI:** 10.21142/2523-2754-1204-2024-220

**Published:** 2024-11-23

**Authors:** Ebingen Villavicencio Caparó, María Elisa Asitimbay Jerez

**Affiliations:** 1 Carrera de Odontologia, Universidad Catolica de Cuenca. Cuenca, Ecuador. maria.asitimbay.67@est.ucacue.edu.ec Universidad Católica de Cuenca Carrera de Odontologia Universidad Catolica de Cuenca Cuenca Ecuador; 2 Doctorado en Salud Colectiva Ambiente y Sociedad, Universidad Andina Simon Bolivar, sede Quito. Quito, Ecuador. evillavicencioc@ucacue.edu.ec Universidad Andina Simón Bolívar Doctorado en Salud Colectiva Ambiente y Sociedad Universidad Andina Simon Bolivar Quito Ecuador evillavicencioc@ucacue.edu.ec

**Keywords:** prevalence, DMFT index, oral health, Ecuador, social epidemiology, prevalencia, índice CPOD, salud oral, Ecuador, epidemiología social

## Abstract

**Objective:**

To analyze the relationship between caries experience and social insertion in 12-year-old schoolchildren from an urban population in the Ecuadorian highlands. Materials and

**Methods:**

A cross-sectional descriptive design was conducted in 2023 with 12-year-old students in the Bellavista parish, Cuenca, Ecuador. Caries experience was measured using the severity levels of the WHO (World Health Organization) DMFT index. At the same time, social insertion was determined through the INSOC (Social Insertion) survey. The total population included 458 data points for the caries experience evaluation phase and 422 for the social insertion phase. Pearson's chi-square and Mann-Whitney U tests were applied.

**Results:**

A percentage of 60.7% of schoolchildren had current caries. Regarding caries experience, 43.7% showed a shallow level, and 11,8% a very high level. Regarding sex, no statistically significant difference was found when comparing DMFT levels (X2, p=0.393). Regarding social insertion, schoolchildren came from the working-class and sub-salaried employee strata with 27.5% and 20.9%, respectively. No significant association was found between caries experience and social insertion in this population (X2 p=0.459). The social insertion stratum with the highest frequency of high DMFT levels was that of the children of migrants, with 27.27%.

**Conclusion:**

No significant association was found between caries experience and social insertion.

## RESUMEN

**Objetivo:**

Analizar la experiencia de caries con la inserción social en escolares de 12 años en una población urbana de la sierra ecuatoriana.

**Materiales y métodos:**

Diseño descriptivo transversal, realizado en 2023, con estudiantes de 12 años en la parroquia Bellavista (Cuenca, Ecuador). Se midió la experiencia de caries utilizando los niveles de severidad del índice de CPOD de la OMS, mientras que la inserción social se determinó a través de la encuesta INSOC. La población total incluyó 458 datos para la fase de evaluación de la experiencia de caries y 422 para la fase de inserción social. Se aplicaron las pruebas de chi-cuadrado de Pearson y U de Mann-Whitney.

**Resultados:**

El 60,7% de los escolares presentaron caries actuales. En cuanto a la experiencia de caries, el 43,7% mostró un nivel muy bajo y el 11,8%, un nivel muy alto. En relación con el sexo, no se encontró diferencia estadísticamente significativa al comparar los niveles de CPOD (x^2^, p = 0,393). En cuanto a la inserción social, los escolares provienen de estrato trabajador obrero y de empleados subasalariados con un 27,5% y un 20,9%, respectivamente. No se encontró asociación significativa entre la experiencia de caries y la inserción social en esta población (x^2^ p = 0,459). El estrato de inserción social con mayor frecuencia del nivel alto de CPOD fue el de los hijos de migrantes, con un 27,27%.

## INTRODUCTION

Latin American social medicine is a counter-hegemonic current within epidemiology and public health that dates back to the 1970s. One of its creators is Dr. Jaime Breilh Paz y Miño, who, in the year 2023, published a book edited by the University of Oxford on critical epidemiology and the health of the peoples of Latin America ^1^; the document above was praised by the editor of the Lancet journal of the United Kingdom ^2^. The present study aims to apply part of this methodology to the study of dental caries.

Dental caries is one of the most prevalent oral conditions worldwide, manifesting through the demineralization of the hard tissues of the tooth ^3^^,^^4^. Its etiology is multifactorial and non-transmissible. Several epidemiological studies have identified oral microbiota, sugar consumption, and the quality of oral hygiene during tooth eruption as critical factors in its etiology ^5^^,^^6^. Martins et al., in their systematic review in 2023, mentioned that dental caries affect not only oral health but also the quality of life of people ^7^.

The World Health Organization (WHO) highlights the close relationship between a healthy body and a healthy mouth, recognizing that quality of life and oral health are essential for general wellbeing and individual health ^8^. Several studies have identified schoolchildren as a population vulnerable to the presence of dental caries. This condition not only affects their ability to eat, smile, and speak and generates pain and discomfort. These problems negatively influence their self-esteem, causing feelings of embarrassment and affecting their emotional wellbeing. In addition, poor oral health can affect school performance, increasing students' non-attendance at educational institutions. Therefore, adequate oral care is crucial to promoting a healthy life ^6^^,^^8^^,^^9^. 

To evaluate dental health, the WHO recommends using the caries experience index (DMFT), which has been used for over 70 years. This index is used for permanent dentition, and its acronym stands for (T) teeth, (D) decayed, (M) missed, and (F) filled due to caries ^10^. The sum of these three conditions determines the DMFT index individually. In contrast, the application of this index at the community level is determined by the sum of decayed, missing, and filled teeth divided by the number of individuals evaluated ^11^. The WHO classifies the DMFT index into five levels of severity: very low (0 to 1.1), low (1.2 to 2.6), moderate (2.7 to 4.4), high (4.5 to 6.5), and very high (6.6 or > 6.6). In addition, it is the most widely used index in epidemiological studies to assess the oral health status of a community, considering risk factors such as age, gender, oral hygiene, socioeconomic status, and knowledge about oral health ^10^^-^^12^. 

According to the WHO, dental caries affects between 60% and 90% of school children, especially in developing countries ^13^. In Ecuador, the Ministry of Public Health conducted a national epidemiological study of oral health in school children under 15 years of age, where it was observed that the prevalence of caries was 60.8% in 12-year-old children, about the DMFT index, a moderate severity of 2.95 was evidenced ^14^. An epidemiological study of oral health in 12-year-old children in Quito-Ecuador showed a caries prevalence of 60.3%, with a low severity level of 1.6 ^15^. Another epidemiological study conducted in Cuenca in 2016 in 12-year-old schoolchildren with a sample of 3407 reported a caries prevalence in both urban and rural parishes of 66.7%. It showed a caries prevalence of 40.9% in the urban parish of Bellavista, indicating a difference between urban and rural parishes ^16^. 

Classical epidemiology considers socioeconomic level one of the leading social determinants of health. However, in South America, with the birth of social medicine, another way of analyzing the social determination of health has emerged. One of the critical aspects of this approach is the social insertion of the families to which schoolchildren belong. According to this theory, each social class has a way of life determined by the position people occupy within the social relations of production or labor, by the relationship they have with the ownership of the means of production, the role they play in work and the proportion of the wealth they receive for their participation in production. This analysis makes it possible to avoid comparisons based on indicators of access to consumption or monetary poverty, which tend to hide the power relations between these social groups ^17^. In 2012, a study was conducted in Colombia using this new methodology, which showed different epidemiological profiles among the various social class fractions ^18^. 

In the case of school-age children, oral health is influenced by several factors, such as the oral hygiene of the parents and the children themselves, as well as the educational level of the parents ^19^^,^^20^. However, these studies do not delve into families' social class and social insertion. A systematic review showed that the lower the parents' educational level, the higher the risk of dental caries in their children ^21^. However, parents' educational level does not arise spontaneously; instead, it is a consequence of the historical social determination of class, gender, and race ^1^. The lack of knowledge about oral health prevention measures contributes to increased diseases in low-income and high-income countries that do not invest in health promotion and prevention ^8^. The social determination of health, a theory of Latin American social medicine, explains that some countries are low-income and others high-income due to the hegemony exercised by high-income countries over those colonized in the Middle Ages ^22^. Critical epidemiology states that the global economic model is determined by the countries' health systems, affecting some differently than others; this inequality is expressed in a result called inequity in health ^1^.

Since it has been evidenced that dental caries has a lower prevalence in the parish mentioned above, it is sought to find a differentiated result of caries among the different social class fractions that inhabit this community. Therefore, the present article aims to analyze the caries experience with the social insertion of the families of 12-year-old school children from an urban population of the Ecuadorian highlands.

## MATERIALS AND METHODS

An epidemiological study of cross-sectional descriptive design was conducted in 2023 on schoolchildren from the parish of Bellavista in Cuenca, Ecuador. Compliance with the ethical principles established in the Declaration of Helsinki was guaranteed, and approval was obtained from the Human Research Ethics Committee of the Catholic University of Cuenca through Resolution CEISH - UCACUE - 013, issued on May 18, 2023. Informed consent was obtained from the parents or legal representatives and the students' assent. The macro-project addressed the relationship between social determinants and oral diseases in 12-year-old schoolchildren from the Bellavista parish educational institutions in Cuenca, Ecuador.

The diagnosis of dental caries was made using the diagnostic recommendations of the DMFT index, which forms part of the sections of the epidemiological record used in this study. The social insertion survey (INSOC) was applied to evaluate the social insertion of the schoolchildren.

• The dependent variable was caries experience, defined as the proportion of schoolchildren currently affected by caries who had a filling or extraction for the same reason. This measure was averaged across the study population and categorized according to severity level ^12^: very low (0 to 1.1); low (1.2 to 2.6); moderate (2.7 to 4.4); high (4.5 to 6.5) and very high (6.6 or > 6.6).

• The independent variable was social insertion, classified according to the parents' occupation or the schoolchildren's legal representatives. This variable was classified into two main categories, which include social class sub-fractions:

- Owners of the means of production (entrepreneur and micro-entrepreneur, subsistence producers, landowning owner).

- Labor force (executive worker, professional or senior technical worker, administrative employee, laborer worker, domestic employee, underpaid employee).

Two class sub-fractions not considered in the original instrument were incorporated: unemployed and migrant, given that the phenomenon of migration in Ecuador is an essential characteristic of this country's economy ^23^. 

• The covariate of this study was sex, dividing the participants into two categories: female and male.

### Population

This study involved 458 students aged 12 from various schools in the urban sector of the Bellavista parish. The distribution of the population was as follows: 26.20% of the participants were from the Federico Proaño school, 26.63% from the Tres de Noviembre school, 22.92% from the Julio María Matovelle school, 17.46% from the Francisca Dávila school, and 6.76% from the Brummel school (Table 1). 

**Table 1:** Distribution of the Population

SCHOOL	BRUMMEL		FEDERICO PROAÑO		FRANCISCA DAVILA		JULIO MARIA MATOVELLE		TRES DE NOVIEMBRE		Total	
	n	%	n	%	n	%	n	%	n	%	n	%
FEMALE	18	58.06	67	55.83	55	68.75	45	42.86	71	58.20	256	55.90
MALE	13	41.94	53	44.17	25	31.25	60	57.14	51	41.80	202	44.10
Total	31	6.76	120	26.20	80	17.46	105	22.92	122	26.63	458	100

Schoolchildren with systemic diseases who had fixed orthodontic appliances, under medication treatment for more than three months or did not respond to the social insertion survey were excluded. After this process, a final population was formed for the Caries Experience phase of 458 data and the social insertion phase of 422 data collected from schoolchildren from the Bellavista parish of Cuenca.

### Calibration

Professionals from the odontology area trained the examiners to ensure accuracy in data collection. The training consisted of several stages: the first consisted of an initial talk on the sections of the epidemiological record and its correct filling out, the second was to analyze 20 samples of teeth with various conditions of caries lesions and to evaluate the concordance with an expert, using the Kappa index. The observed data were recorded according to the WHO coding of the state of the dentition ^24^. Only examiners with a Kappa concordance >0.8 were admitted for the study. For the calibration of the surveys for social insertion, a pilot test was performed with patients from the university dental clinic, and concordance was assessed with an expert in the same way as for caries.

### Examination

The clinical examination was carried out in classrooms of the educational institutions, using natural lighting, between 8 am and 10:50 am before recess. During the intraoral evaluation, the students remained seated to facilitate access and comfort. The information was collected employing epidemiological records, applying the WHO classification of clinical findings of the DMFT index in permanent dentition ^24^. For social insertion, the questionnaire was applied with the parameters established by its author ^17^. For the clinical examination, instruments sterilized one night before were used. Each examiner had a note-taker responsible for filling out the cards.

The data collected were recorded on the cards and stored in the EPI INFO software version 7.2.6.0. Subsequently, 100% of the information was quality-controlled, checking that what was written on the card matched what was recorded in the database. Once it was verified that there were no errors, the data were exported to SPSS V.29 software for statistical analysis.

### Statistical analysis

The data obtained on DMFT were analyzed descriptively using the mean and standard deviation; then, this variable was classified into levels of the degree of severity and reported through frequencies, percentages, and graphs. Since the latter is an ordinal qualitative variable, Pearson's Chi-square test was used with a margin of error ≤ 0.05 to evaluate the association with the social insertion variable.

The prevalence of caries was calculated following this rule: the carious pieces component was categorized into two groups: patients with 0 caries were considered healthy, and all others were considered diseased. In this categorization, missing and filled teeth were not included. To compare caries experience according to sex in the different social insertion groups, the Mann-Whitney U test was used, with a reliability of 95%.

## RESULTS

In Cuenca, Ecuador, the political-territorial division comprises 36 parishes that comprise the city's administrative structure. Of these parishes, 15 are located in the urban area and 21 in the rural area, reflecting a diverse geographic distribution and demographic characteristics. The Bellavista parish is located in the city's urban area, is home to 8% of the total population, and has an area of 499.27 hectares. It is home to 6 publicly managed educational institutions, 5 of which agreed to participate in the study.

### Distribution of the study population:

This study focused on analyzing 458 schoolchildren. The study population was distributed among five educational institutions: Brummel, Federico Proaño, Francisca Dávila, Julio María Matovelle, and Tres de Noviembre (Table 1).

### Prevalence of dental caries in the total population:

**Figure 1:**
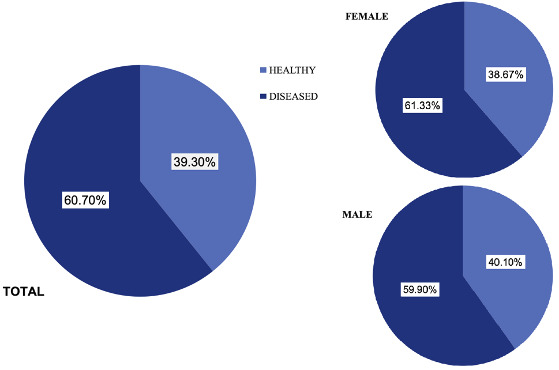
Prevalence of caries in the study population.

In the study, most of the population was observed to have caries lesions (Figure 1).

### Levels of severity of dental caries experience in schoolchildren:

In the analysis of the DMFT levels of the 458 schoolchildren, it was observed that 43.7% of schoolchildren present a very low level, indicating a number between 0 and 1.1 teeth with caries experience. A moderate level of DMFT was observed in 22.7% of the schoolchildren, while 16.4% had a low level of DMFT. On the other hand, 11.8% of schoolchildren have a high level of DMFT, and 5.5% reach a very high level. These results indicate that, although most schoolchildren have a very low level of DMFT, there is a considerable group with a moderate level.

### Levels of caries experience according to sex

**Figure 2:**
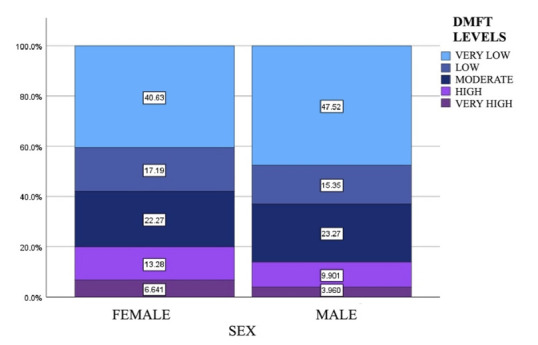
DMFT levels according to sex

No statistically significant difference was found when comparing DMFT levels between both sexes (X2 p=0.393). The female sex had a higher frequency of high and very high levels of DMFT (13.28% and 6.64%, respectively). On the other hand, males had a higher frequency of low (15.35%) and very low (47.52%) DMFT levels (Figure 2).

DMFT = permanent dentition index of decayed, missing, and filled teeth.

### Average caries experience of the study population:

The average caries experience of the total population of school children is 2.41 (±2.33), which is classified as low DMFT. Regarding gender, the female group presents an average of 2.59 (±2.48), also classified as low. In the male group, the average is 2.19 (±2.12), also classified as low DMFT. No significant difference was found in the Mann-Whitney U test (p= 0.139).

### Social insertion of the population studied.

Regarding social insertion, it was observed that most schoolchildren come from families whose parents or legal representatives work in working-class and technical professional occupations. The social class fractions are Laborer Worker (27.5%), Underpaid Employee (20.9%), and Professional or Senior Technical Worker (19.4%), representing 67.8% of the total population surveyed, which means that it is a sample of a middle-class group of people. Only a minority of the schoolchildren come from families with owner occupations, such as Entrepreneur and Micro-entrepreneur (1.9%) and Landowning Farmer (1.2%).

### Association between caries experience and social insertion

**Figure 3:**
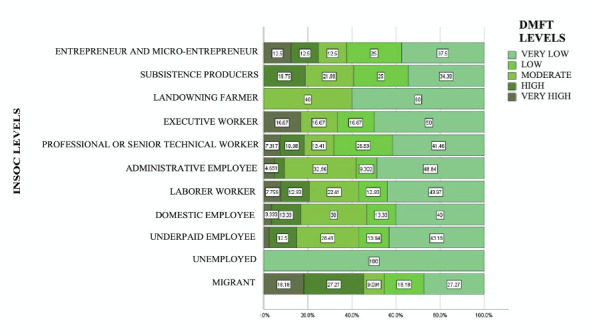
Levels of DMFT and Social Insertion

No association was found between caries experience and social insertion (X2 p=0.459). Schoolchildren belonging to the Migrant social insertion level presented a higher frequency of high and very high levels of DMFT. In contrast, schoolchildren in the Entrepreneur and Micro-entrepreneur social insertion levels presented low and very low levels of DMFT (Figure 3).

DMFT = permanent dentition index of decayed, missing, and filled teeth. INSOC = Social Insertion.

### Comparison of caries experience by sex in social insertion groups:

At most levels of social insertion, the female sex presented a higher average DMFT relative to males. Notable exceptions were observed at the level of managerial workers, where the female sex presented a high average DMFT. Caries experience showed more significant influence in certain groups, such as the managerial and migrant worker level, with DMFT being more prevalent in the female sex. The Mann-Whitney U test indicated that there was no statistically significant difference (p > 0.05) (Table 2).

**Table 2:** Comparison of the DMFT index according to sex in the social insertion groups

INSOC	N	DMFT				Mann Whitney
		FEMALE		MALE		U
		Mean	Standard deviation	Mean	Standard deviation	p
Entrepreneur and Micro-Entrepreneur	8	3.50	4.37	2.50	3.54	0.86
Subsistence Producers	32	2.44	1.92	2.50	2.10	1.00
Landowning Farmer	5	0.00	-	1.75	2.06	1.00
Executive Worker	6	12.00	-	1.00	1.41	0.33
Professional Or Senior Technical Worker	82	2.76	2.84	2.03	2.22	0.21
Administrative Employee	43	2.30	1.98	2.06	1.88	0.68
Laborer Worker	116	2.53	2.37	2.54	2.50	0.98
Domestic Employee	30	2.62	2.33	2.18	1.91	0.68
Underpaid Employee	88	2.42	2.19	2.03	1.73	0.57
Unemployed	1	1.00	-	-	-	N.A.
Migrant	11	4.50	3.15	3.00	2.45	0.54

DMFT = permanent dentition index of decayed, missing, and filled teeth.

INSOC = Social insertion.

## DISCUSSION

The study was conducted in an urban area of the City of Cuenca, Ecuador, evaluating caries experience and social insertion association. For the evaluation of dental caries, the criteria of decayed, missing, and filled teeth for permanent dentition (DMFT) were used ^24^, while the INSOC survey was used for social insertion ^17^. In the latest reports on oral health, the WHO emphasizes that oral health is an integral and essential part of general health and determines each person's quality of life ^26^. 

The present study observed a high prevalence of dental caries of 60.70%. At a global level, the WHO highlights that between 60% and 90% of school children present dental caries ^13^. These findings are comparable with the study conducted in Costa Rica by Ramirez and Gomez ^27^, who reported a prevalence of 59.7%. Similarly, another epidemiological study conducted by the Ministry of Public Health on 12-year-old schoolchildren nationwide in Ecuador by Raza et al. ^14^ reported a prevalence of 60.8%. Similarly, an epidemiological study in the capital city (Quito) by Michel et al. ^15^ reported a caries prevalence of 60.3%. In contrast to these results, the epidemiological study carried out in the city of Cuenca, in the same parish, by Villavicencio, Reinoso, and Encalada ^16^ indicated a prevalence of 40.9%, significantly lower than the present study. This discrepancy could be due to the sample size for this parish, which was smaller than the sample used in the current study.

Regarding DMFT levels according to sex, the present study showed that 40.60% of the female participants had a very low level of DMFT. In comparison, only 6.41% had a very high caries experience. On the other hand, 47.52% of the male sex was in the very low level, and only 3.96% in the very high level. It compared with this study presented opposite results to those obtained by Fernandez, Romo, and Cabrera ^28^, who pointed out that the female sex has a lower percentage in the low level with 9. Similarly, the results of the male sex also differed from the study in the Bellavista parish, which showed a higher percentage in the very high level (29.6%) and a lower percentage in the very low level (12%). The population size could explain these differences in the current study, which consisted of 458 participants compared to 279 in the above study.

Regarding the DMFT index, the study population presented an average of 2.41, corresponding to a low severity level, results that were similar to those obtained by Martínez, Piedra, Paladines ^29^ in 12-year-old schoolchildren in an urban parish of the same city where the present study was carried out, showing a very low level of caries. In contrast, the findings of Orellana, Herbas, Calizaya, and Mamani ^11^ in Bolivia showed that the schoolchildren presented moderate severity, with a caries experience index 2.7. On the other hand, the study by Fernández, Núñez, and Díaz ^30^, carried out on 12-year-old schoolchildren in Chile, reported a moderate level of caries with an average of 3.15. The difference between the findings of the present study and those of the research mentioned above could be due to the demographic characteristics because this study covered both rural and urban areas of Chile.

This study revealed that the majority of the schoolchildren examined were predominantly in the labor force category, with the highest percentage in the sub-fraction of laborer worker (27.5%), followed by the sub-fraction of sub-salaried employee (20.9%) and, in third place, by the sub-fraction of professional or higher technical worker (19.4%). However, when analyzing the relationship between the levels of the DMFT index and social insertion, it was observed that schoolchildren in the migrant sub-fraction had a high (27.27%) and very high (18.18%) level of caries severity. In contrast, schoolchildren in the sub-fraction of entrepreneurs and micro-entrepreneurs, classified as owners of the means of production, presented a low (25%) and very low (37.5%) level of caries severity. Previous research, such as that of Miranda et al. ^31^, indicates that unemployment and low parental income are associated with caries in their children. Similarly, Pavón et al. ^32^ point out that low family income, education, and cultural level contribute to the risk factors for developing dental caries due to the difficulty in accessing oral health promotion and prevention.

Therefore, it is necessary to consider the research of Vaccaro et al. ^26^, who suggest that public health policies should focus on oral hygiene education from an early age, promoting habits such as regular tooth brushing and flossing. They also emphasize the need to improve oral health services, especially in vulnerable and low-income populations, to ensure adequate preventive care and necessary treatment; this strategy would reduce the incidence of caries in schoolchildren of the migrant sub-fraction.

## LIMITATIONS

The main limitation of this study was that the Cartesian positivist method only allows evaluating concordances of variables in space and time; a new form of critical epidemiology would be an improvement to this study and perhaps would better explain the trends found in the health indicators of people. This information can probably yield more revealing results if analyzed under the critical epidemiology methodology proposed by Jaime Breilh, which takes into account a meta-critical approach from the viewpoint of the city's socio-historical conformation and the contradictions of the social classes in their ways of life and individual lifestyles ^1^.

Another limitation of this study was the loss of a small part of the study population because several students withdrew from the schools included in the study, changed to other educational institutions, or moved out of the country. The reduction in the number of participants does not affect the statistics of this study since we worked with the total population and not with a sample.

## CONCLUSION

No significant association was found between caries expression and social insertion in this population. This study found a high prevalence of dental caries in 12-year-old schoolchildren, according to the DMFT index criteria, with a generally very low level of caries severity in both sexes. However, females presented a higher experience of dental caries than males. Regarding social insertion, many schoolchildren belong to families with a social insertion level of workers and technical professionals. Schoolchildren with a social insertion level of migrants present high and very high levels of caries severity.
